# Transforming growth factor β1-induced astrocyte migration is mediated in part by activating 5-lipoxygenase and cysteinyl leukotriene receptor 1

**DOI:** 10.1186/1742-2094-9-145

**Published:** 2012-06-26

**Authors:** Xue-Qin Huang, Xia-Yan Zhang, Xiao-Rong Wang, Shu-Ying Yu, San-Hua Fang, Yun-Bi Lu, Wei-Ping Zhang, Er-Qing Wei

**Affiliations:** 1 Department of Pharmacology, Key Laboratory of Medical Neurobiology of Ministry of Health of China, Zhejiang Province Key Laboratory of Neurobiology, Zhejiang University School of Medicine, 866 Yuhangtang Road, Hangzhou, 310058, China

**Keywords:** Transforming growth factor-β1, Cysteinyl leukotriene, Cysteinyl leukotriene receptor, 5-lipoxygenase, Astrocyte, migration, Glial scar

## Abstract

**Background:**

Transforming growth factor-β1 (TGF-β1) is an important regulator of cell migration and plays a role in the scarring response in injured brain. It is also reported that 5-lipoxygenase (5-LOX) and its products, cysteinyl leukotrienes (CysLTs, namely LTC_4_, LTD_4_ and LTE_4_), as well as cysteinyl leukotriene receptor 1 (CysLT_1_R) are closely associated with astrocyte proliferation and glial scar formation after brain injury. However, how these molecules act on astrocyte migration, an initial step of the scarring response, is unknown. To clarify this, we determined the roles of 5-LOX and CysLT_1_R in TGF-β1-induced astrocyte migration.

**Methods:**

In primary cultures of rat astrocytes, the effects of TGF-β1 and CysLT receptor agonists on migration and proliferation were assayed, and the expression of 5-LOX, CysLT receptors and TGF-β1 was detected. 5-LOX activation was analyzed by measuring its products (CysLTs) and applying its inhibitor. The role of CysLT_1_R was investigated by applying CysLT receptor antagonists and CysLT_1_R knockdown by small interfering RNA (siRNA). TGF-β1 release was assayed as well.

**Results:**

TGF-β1-induced astrocyte migration was potentiated by LTD_4_, but attenuated by the 5-LOX inhibitor zileuton and the CysLT_1_R antagonist montelukast. The non-selective agonist LTD_4_ at 0.1 to 10 nM also induced a mild migration; however, the selective agonist N-methyl-LTC_4_ and the selective antagonist Bay cysLT2 for CysLT_2_R had no effects. Moreover, CysLT_1_R siRNA inhibited TGF-β1- and LTD_4_-induced astrocyte migration by down-regulating the expression of this receptor. However, TGF-β1 and LTD_4_ at various concentrations did not affect astrocyte proliferation 24 h after exposure. On the other hand, TGF-β1 increased 5-LOX expression and the production of CysLTs, and up-regulated CysLT_1_R (not CysLT_2_R), while LTD_4_ and N-methyl-LTC_4_ did not affect TGF-β1 expression and release.

**Conclusions:**

TGF-β1-induced astrocyte migration is, at least in part, mediated by enhanced endogenous CysLTs through activating CysLT_1_R. These findings indicate that the interaction between the cytokine TGF-β1 and the pro-inflammatory mediators CysLTs in the regulation of astrocyte function is relevant to glial scar formation.

## Background

Glial scar formation is a critical event in repair responses after injury of the central nervous system (CNS) [1,2]. The glial scar is a complex of cellular components and mainly consists of reactive astrocytes (undergoing proliferation and morphological changes). Following focal CNS injury, reactive astrocytes migrate towards the lesion and then organize into a densely packed glial scar [1,2]. As the key step of glial scar formation, astrocyte migration is regulated by various factors [3-5], among which transforming growth factor-β (TGF-β) is known as an important regulator [5,6].

TGF-β, a family of multifunctional cytokines, regulates a broad diversity of physiological and pathological processes, including wound healing, inflammation, cell proliferation, differentiation, migration and extracellular matrix synthesis [7-10]. TGF-β1 is an important mediator in the pathogenesis of several disorders in the CNS, such as in the organization of a glial scar in response to injury and in several neurodegenerative disorders [7,11,12]. After CNS injury, elevated TGF-β levels in astrocytes have been shown to induce astrocytic scar formation [13], and are also associated with ischemic brain injury [14,15].

On the other hand, cysteinyl leukotrienes (CysLTs, namely LTC_4_, LTD_4_, and LTE_4_), the 5-lipoxygenase (5-LOX, EC 1.13.11.34) metabolites of arachidonic acid [16], are bioactive lipid mediators that modulate immune and inflammatory responses [16-19] through activating their receptors, CysLT_1_R and CysLT_2_R [17,20,21]. In the rat brain, 5-LOX is activated and the production of CysLTs is enhanced after focal cerebral ischemia, resulting in neuronal injury and astrocyte proliferation (astrocytosis). This post-ischemic astrocytosis is associated with up-regulated CysLT_1_R and CysLT_2_R [22-26]. The CysLT_1_R antagonist pranlukast attenuates post-ischemic astrocytosis and glial scar formation in the chronic phases of focal cerebral ischemia in mice and rats [25,27,28]. This effect suggests that CysLT_1_R mediates CysLT-induced astrocytosis and glial scar formation in response to *in vivo* ischemic injury. In primary astrocyte cultures, CysLTs are released after oxygen-glucose deprivation-induced ischemic injury, and the resultant activation of CysLT_1_R mediates astrocyte proliferation [29,30]. These findings imply that the endogenously released CysLTs might play an autocrine role in the induction of astrocytosis and resultant glial scar formation through activating CysLT_1_R.

However, whether CysLT_1_R mediates astrocyte migration in the process of glial scar formation needs investigation. In the periphery, CysLT_1_R mediates migration in many types of cells, such as monocytes [31], dendritic cells [32], monocyte-derived dendritic cells [33], vascular smooth muscle cells [34], intestinal epithelial cells [35] and endothelial cells [31,34-36]. Therefore, CysLT_1_R may also be an inducer of astrocyte migration, but many other factors have been reported to be potent inducers, such as TGF-β1 [37,38]. Thus, there may be interactions between CysLT_1_R and other regulators (for example, TGF-β1). TGF-β1 up-regulates CysLT_1_R expression and increases the production of CysLTs in several cell types such as hepatic stellate cells [39] and bronchial smooth muscle cells [37]. Based on these findings, it is possible that the regulatory role of TGF-β1 in astrocyte migration may be mediated by enhanced production of CysLTs *via* CysLT_1_R activation. To clarify this possibility, in the present study, we investigated the interactions between TGF-β1 and 5-LOX/CysLT_1_R in astrocyte migration.

## Methods

### Primary cultures of rat astrocytes

Primary astrocytes were isolated from the cerebral cortex of newborn Sprague–Dawley rats within 24 h as described previously [30,40]. In brief, the cortices were digested with 0.25% trypsin and plated into poly-L-lysine-coated flasks. Cells were cultured in high-glucose DMEM (Gibco, Grand Island, NY, USA) supplemented with 10% fetal bovine serum (FBS), 2 mM glutamine, 100 units/mL penicillin and 100 μg/mL streptomycin at 37°C in a humidified atmosphere of 95% air/5% CO_2_. After incubation for 11 to 14 days, the confluent cultures were shaken overnight at 260 rpm at 37°C, and the adherent cells were trypsinized and re-seeded in the growth medium. More than 95% of the cells were astrocytes as confirmed by immunofluorescence staining for glial fibrillary acidic protein (GFAP).

All animal experiments were carried out in accordance with the National Institutes of Heath Guide for the Care and Use of Laboratory Animals. We made every effort to minimize the number of animals used and their suffering. The experimental protocols were approved by the Ethics Committee of Laboratory Animal Care and Welfare, School of Medicine, Zhejiang University.

### Cell migration (wound healing) assay

Astrocytes were grown to confluence in 24-well plates and starved in serum-free DMEM for 24 h. The monolayer cells were manually scratched with a 20-μl pipette tip to create an extended and definite scratch in the center of the dish with a bright and clear field. The detached cells were removed by washing with phosphate-buffered saline (PBS). DMEM containing 1% FBS with or without TGF-β1 (PeproTech Inc, Rocky Hill, NJ, USA) was added to each dish. In some experiments, 1 ng/ml TGF-β1 was added to each dish for 30 minutes before treatment with LTD_4_ (Sigma-Aldrich Co., St Louis, MO, USA) or N-methyl LTC_4_ (NMLTC_4_, a metabolically stable LTC_4_ mimetic; Cayman Chemical Co., Ann Arbor, MI, USA). Cells were pretreated with the following inhibitor and antagonists: zileuton (0.01 to 5 μM, a 5-LOX inhibitor; Gaomeng Pharmaceutical Co., Beijing, China), montelukast (0.01 to 5 μM, a selective CysLT_1_R antagonist; Merck & Co., Inc., Whitehouse Station, NJ, USA), and Bay cysLT2 (0.01 to 5 μM, a selective CysLT_2_R antagonist; a kind gift from Dr. T. Jon Seiders of Amira Pharmaceuticals, Inc., San Diego, CA, USA) for 30 minutes, and then incubated with TGF-β1 for 24 h. Images of migratory cells from the scratch boundary were acquired at 0 and 24 h under a light microscope with a digital camera.

To continuously monitor migration time-course in live astrocytes, astrocytes were plated in 35-mm dishes and grown to confluence, and then the cells were scratched and treated with LTD_4_ or/and TGF-β1 as described above. The movements of live astrocytes was traced under an inverse videomicroscope (Olympus IX81, Olympus Corp., Tokyo, Japan), and the wound was photographed at 0, 6, 12, 18 and 24 h.

The wounded areas were analyzed with ImageTool 2.0 software (University of Texas Health Science Center, San Antonio, TX, USA). The wound healing effect is determined as the initial scratch area (0 h) after wounding minus the scratch area after treatment for 24 h, or 6, 12, 18 and 24 h (live astrocytes), and reported as percentages of control values. Moreover, some astrocyte samples seeded on coverslips were visualized by GFAP immunofluorescence staining 24 h after scratching as the typical images.

### Cell proliferation assay

To measure astrocyte proliferation, carboxyfluorescein diacetate succinimidyl ester (CFSE) green fluorescent dye (Invitrogen Corp., Carlsbad, CA, USA) dilution assay was performed according to the manufacturer’s instructions and the reported method [41-43]. Briefly, astrocytes were grown to confluence in six-well plates and starved in serum-free DMEM for 24 h, then the cells were washed twice with PBS and incubated in 5 μM CFSE in PBS for 15 minutes at 37°C, and subsequently washed twice with PBS. Then DMEM containing 1% FBS with or without TGF-β1 or LTD_4_ was added to each plate. In some experiments, 1 ng/ml TGF-β1 was added to each plate for 30 minutes before treatment with LTD_4_. The cells were harvested at 24 h, and subjected to fluorescence activated cell sorting using the FC500MCL flow cytometer (Beckman Coulter, Inc., Brea, CA, USA). Proliferation was measured by loss of CFSE dye.

### CysLT_1_ receptor knockdown by small interfering RNA (siRNA)

RNA duplexes of 21 nucleotides specific for rat CysLT_1_R sequences were chemically synthesized, together with a non-silencing negative control siRNA. The CysLT_1_R siRNA sense sequence was: 5′-CAG CCU UCC AAG UAU ACA UTT-3′ and anti-sense: 5′-AUG UAU ACU UGG AAG GCU GTT-3′; the non-silencing control siRNA sense: 5′-UUC UCC GAA CGU GUC ACG UTT-3′ and anti-sense: 5′-ACG UGA CAC GUU CGG AGA ATT-3′ (GenePharma Co., Shanghai, China). Transfection of siRNA duplexes was performed according to the manufacturer’s instructions. Briefly, astrocytes were seeded on the day before transfection using an appropriate medium with 10% FBS without antibiotics. They were transiently transfected with CysLT_1_R siRNA or negative control siRNA (100 nM) for 6 h using Lipofectamine™ 2000 (Invitrogen, USA). After the transfected cells were incubated for 48 h, they were treated with LTD_4_ or TGF-β1 for cell migration assay.

### Reverse transcription-polymerase chain reaction (RT-PCR)

At the end of the experiments, total RNA was extracted from the cultured astrocytes using Trizol reagent (Invitrogen, USA) according to the manufacturer’s instructions. The cDNA synthesis and PCR reactions were performed as reported previously [29,30]. The PCR primers were: 5-LOX forward 5′-AAA GAA CTG GAA ACA GCT CAG AAA-3′ and reverse 5′-AAC TGG TGT GTA CAG GGG TCA GTT-3′; CysLT_1_R, forward 5′- ATG TTC ACA AAG GCA AGT GG −3′ and reverse 5′-TGC ATC CTA AGG ACA GAG TCA −3′; CysLT_2_R, forward 5′- ACC CCT TCC AGA TGC TCC A −3′ and reverse 5′- CGT GCT TTG AAA TTC TCT CCA −3′; β-actin, forward 5′-AAC CCT AAG GCC AACCGT GAA-3′ and reverse 5′-TCA TGA GGT AGT CTG TCA GGT C-3′; TGF-β1, forward 5′- GAC CGC AAC AAC GCA ATC TA −3′ and reverse 5′- AGG TGT TGA GCC CTT TCC AG −3′.

For cDNA synthesis, 2 μg total RNA was mixed with 1 mM deoxynucleotide triphosphate, 0.2 μg random primer, 20 U RNasin and 200 U M-MuLV reverse transcriptase in 20 μl reverse reaction buffer. The mixture was incubated at 42°C for 60 minutes, and then heated at 72°C for 10 minutes to inactivate the reverse transcriptase.

PCR was performed on an Eppendorf Master Cycler (Eppendorf Scientific, Inc., Westbury, NY, USA) as follows: 1 μl cDNA mixture was reacted in 20 μl reaction buffer containing 1.5 mM MgCl_2_, 0.2 mM deoxynucleotide triphosphate, 20 pM primer and 1 U Taq DNA polymerase. The reaction mixtures were initially heated at 94°C for 2 minutes, then at 94°C for 60 sec, 56°C for 60 sec, and 72°C for 60 sec for 35 cycles and finally stopped at 72°C for 10 minutes. With the exception of TGF-β1, the reaction mixtures were initially heated at 94°C for 2 minutes, then at 94°C for 30 sec, 54°C for 30 sec, and 72°C for 60 sec for 28 cycles and finally stopped at 72°C for 10 minutes. PCR products of 20 μl were separated by 2% agarose gel electrophoresis and visualized by ethidium bromide staining. The density of each band was measured by a UVP gel analysis system (Bio-Rad Laboratories, Hercules, CA, USA). The results are expressed as the ratios to β-actin.

### Western blotting analysis

Astrocytes were washed twice with ice-cold PBS and then lysed for 30 minutes on ice in Cell and Tissue Protein Extraction Solution (Kangcheng Biotechnology Inc., Shanghai, China). The homogenate was centrifuged at 12,000 g for 30 minutes at 4°C, and the supernatant was used. The protein samples (100 μg) were separated by 10% SDS-polyacrylamide gels and then transferred to nitrocellulose membranes (Invitrogen). The membranes were blocked by 10% fat-free milk, and sequentially incubated with the following antibodies: rabbit polyclonal antibody against CysLT_1_R (1:200) [44], CysLT_2_R (1:200) [26,45] or 5-LOX (1:300, (Chemicon International Inc. Temecula, CA, USA) and mouse monoclonal antibody against glyceraldehyde 3-phosphate dehydrogenase (GAPDH) (1:5,000, Kangcheng Biotechnology Inc., Shanghai, China) at 4°C overnight. After repeated wash, the membranes were incubated with anti-rabbit IRDye700DX®-conjugated antibody or anti-mouse IRDye800DX®-conjugated antibody (1:5,000, Rockland Immunochemicals, Inc., Gilbertsville, PA, USA). The immunoblot was analyzed by the Odyssey Fluorescence Scanner (LI-COR Bioscience, Inc., Lincoln, NE, USA). The protein bands were quantified using BIORAD Quantity One software (Bio-Rad, USA). The results are expressed as the ratios to GAPDH.

### Immunofluorescence staining

Astrocytes seeded on coverslips were fixed in cold methanol for 5 minutes, and incubated in 10% normal goat serum for 2 h to block non-specific binding of IgG. Then the cells were reacted with a mouse monoclonal antibody against GFAP (1:500, Millipore Corp., Bedford, MA, USA) and a rabbit polyclonal antibody against CysLT_1_R (1:200, Chemicon, USA) at 4°C overnight. After washing in PBS, astrocytes were incubated with FITC-conjugated goat anti-mouse or Cy3-conjugated goat anti-rabbit antibody (1:200, Millipore, USA) for 2 h at room temperature. Finally, the stained cells were observed under a fluorescence microscope (Olympus BX51, Olympus Corp., Tokyo, Japan). Control coverslips were treated with normal goat serum instead of the primary antibody, and did not show positive immunostaining (data not shown).

### 5-LOX immunocytochemistry

Astrocytes cultured on coverslips were fixed in cold methanol (−20°C) for 5 minutes and incubated for 30 minutes in PBS containing 3% H_2_O_2_ to eliminate endogenous peroxidase activity. Then, cells were incubated for 2 h in PBS containing 10% normal goat serum and incubated at 4°C overnight with rabbit polyclonal antibody against 5-LOX (1:200, Chemicon, USA) as the primary antibody. After three washes with PBS, cells were incubated for 2 h with biotin-conjugated goat anti-rabbit IgG antiserum (1:200) as a second antibody, followed by incubation with avidin-biotin-HRP complex. Finally, the cells were visualized with 0.01% 3, 3′-diaminobenzidine and 0.005% H_2_O_2_ in 50 mM Tris–HCl, pH 7.6. Control coverslips were treated with normal goat serum instead of the primary antibody and they did not show positive immunostaining (data not shown). Then, the cells were examined under the Olympus microscope.

### Measurement of extracellular cysteinyl leukotrienes and TGF-β1

According to the reported method [29,30], astrocytes were seeded into six-well culture plates at 5 × 10^5^ cells/well in 2 ml standard culture medium for 24 h. After culture in DMEM without serum for another 24 h, astrocytes were cultured in DMEM with 1% FBS and stimulated with TGF-β1 (10 ng/mL), various concentrations of LTD_4_ or NMLTC_4_, or vehicle for the designated times. Then, cell-free supernatants were stored at −80°C. The CysLTs (LTC_4_, LTD_4_ and LTE_4_) in astrocyte supernatants were assayed using a commercial CysLT ELISA kit (Cayman Chemical Co., Ann Arbor, MI, USA) according to the manufacturer’s instructions and calculated as pg/mg protein. The TGF-β1 in the supernatants was assayed using a commercial TGF-β1 ELISA kit (Wuhan Boster Biological Technology Co., Ltd., Wuhan, China) according to the manufacturer’s instructions, and calculated as pg/ml.

### Statistical analysis

Data are reported as mean ± S.E.M. Student’s *t-*test and one-way analysis of variance were used to determine the statistical significance of differences between groups. A value of *P* <0.05 was considered statistically significant.

## Results

### TGF-β1- and LTD_4_-induced astrocyte migration

**Figure 1:**
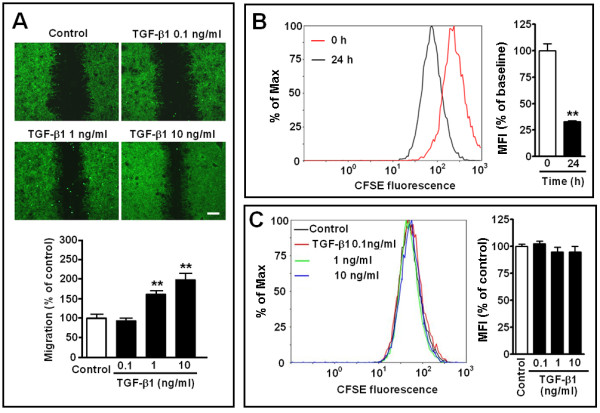
**Effect of TGF-β1 on astrocyte migration and proliferation.** (**A**) Photomicrographs showing migration after treatment with TGF-β1 (0.1 to 10 ng/ml) for 24 h. Scale bar, 400 μm. (**B, C**) Fluorescence intensity was determined by fluorescence activated cell sorting after CFSE labeling at 0 (baseline) and 24 h. Mean fluorescence intensity (MFI) at 24 h reduced compared with baseline (B), but did not change 24 h after treatment with TGF-β1 (0.1, 1 and 10 ng/ml, C). Data are reported as mean ± S.E.M.; *n* = 8 (A), 3 (B) or 9 (C); **P <0.01 compared with control.

First, we confirmed the effect of TGF-β1 on astrocyte migration. TGF-β1 (1 and 10 ng/ml for 24 h) significantly accelerated the migration of astrocytes from the wound edge into the central area in a concentration-dependent manner (Figure 1A). To distinguish the effects on migration and proliferation, we determined whether TGF-β1 affects astrocyte proliferation. The results of CFSE fluorescence intensity showed that astrocyte proliferation did not differ from control level 24 h after exposure to TGF-β1 (0.1, 1 and 10 ng/ml) (Figure 1C) although the assay confirmed astrocyte proliferation at 24 h compared with 0 h (baseline) (Figure 1B).

**Figure 2:**
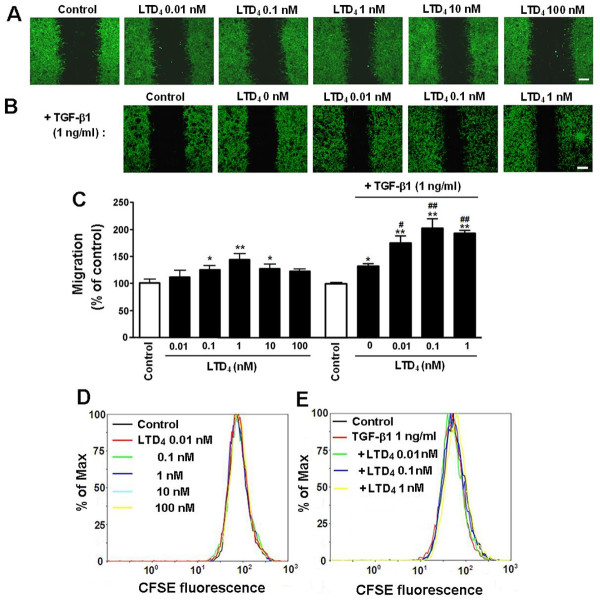
**Effect of LTD**_**4**_**on astrocyte migration and TGF-β1-induced migration and proliferation.** (**A, B**), Photomicrographs showing astrocyte migration 24 h after treatment with LTD_4_ (0.01 to 100 nmol/L) in the absence (A) or presence of TGF-β1 (1 ng/ml, B). Scale bars, 400 μm. (**C**) Data are reported as mean ± S.E.M.; *n* = 8; *P <0.05 and **P <0.01 compared with control, ^#^*P* <0.05 and ^##^*P* <0.01 compared with TGF-β1 alone (LTD_4_ 0). (**D, E**) MFI at 24 h was no significant change 24 h after treatment with LTD_4_ (0.01 to 100 nM, D) alone or combined with TGF-β1 1 ng/ml (E) (*n* = 9 for each group, *P* >0.05).

**Figure 3:**
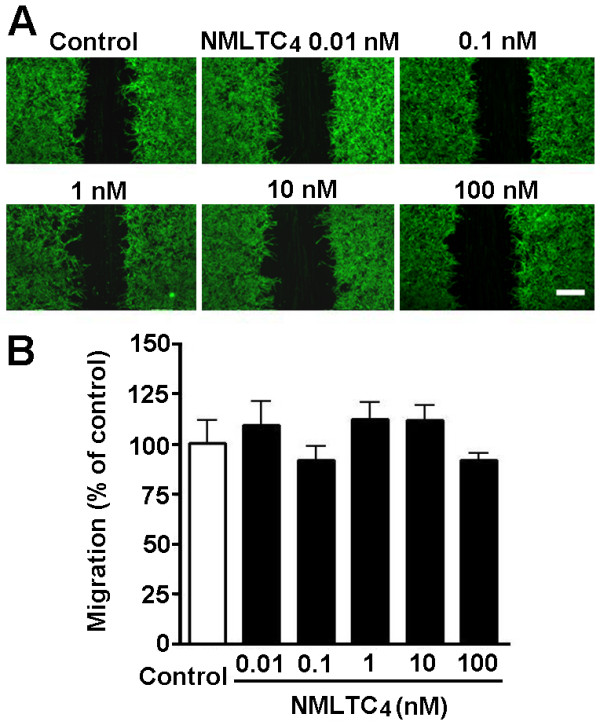
**Effect of NMLTC**_**4**_**on astrocyte migration.** (**A**) Photomicrographs showing astrocyte migration 24 h after treatment with NMLTC_4_ (0.01 to 100 nmol/L). (**B**) Data are reported as mean ± S.E.M.; *n* = 8. Scale bar, 400 μm.

**Figure 4:**
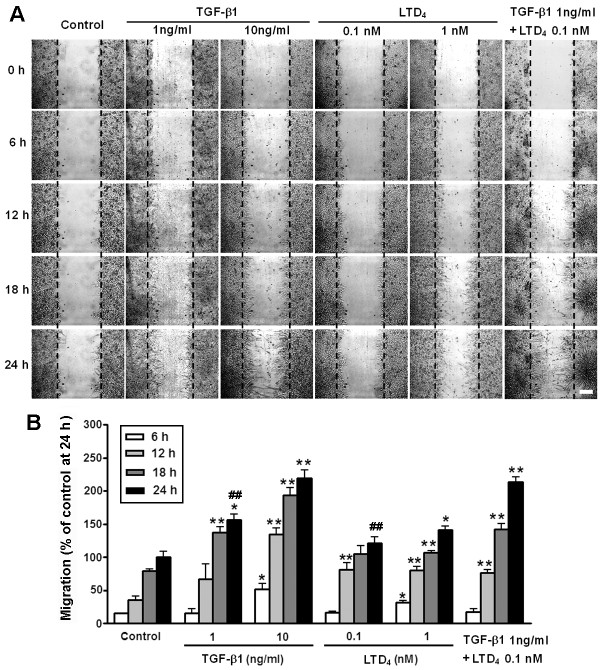
**Time-dependent migration of live astrocytes after exposure to TGF-β1 and LTD**_**4**_**.** Live astrocytes were continuously monitored under a videomicroscope after exposure to TGF-β1 or/and LTD_4_. (**A**) Representative images showing astrocyte migration traced by videomicroscopy at 6, 12, 18 and 24 h after scratching. Scale bar, 200 μm. (**B**) TGF-β1 and LTD_4_ concentration- and time-dependently accelerated migration. When TGF-β1 (1 ng/ml) combined with LTD_4_ (0.1 nM), the effect at 24 h was more potent than that of TGF-β1 or LTD_4_ alone. Data are reported as mean ± S.E.M.; *n* = 3; *P <0.05 and **P <0.01 compared with corresponding time-points of the control; ^##^*P* <0.01 compared with TGF-β1 + LTD_4_ at 24 h.

Next, we determined whether the non-selective agonist LTD_4_ and the CysLT_2_R agonist NMLTC_4_[46] induce astrocyte migration, and LTD_4_ potentiates the TGF-β1 effect. The results showed that LTD_4_ significantly stimulated the migration of astrocytes at 0.1 to 10 nM but not at 0.01 and 100 nM; the maximum migration (141.7 ± 5.0%) was induced by 1 nM LTD_4_ (Figure 2A, C). LTD_4_ (0.01 to 1 nM) also potentiated the effect of the lower concentration of TGF-β1 (1 ng/ml); the migration rates after treatment with 1 ng/ml TGF-β1 were increased from 110.3 ± 5.4% to 175.3 ± 4.8% with 0.01 nM, from 123.5 ± 4.0% to 203.5 ± 5.3% with 0.1 nM, and from 141.7 ± 5.0% to 193.8 ± 2.9% with 1 nM LTD_4_ (Figure 2B, C). LTD_4_ (0.01 to 100 nM) alone (Figure 2D) or combined with TGF-β1 1 ng/ml (Figure 2E) did not affect astrocyte proliferation at 24 h. However, NMLTC_4_ (0.01 to 100 nM for 24 h) did not have any significant effect on astrocyte migration (Figure 3). In addition, to confirm the migration and determine its temporal property, we continuously monitored migration of live astrocytes during 24 h after exposure to LTD_4_ or/and TGF-β1. We found that TGF-β1 (1 and 10 ng/ml) and LTD_4_ (1 nM) gradually accelerated migration during 24 h in a concentration-dependent manner. When TGF-β1 (1 ng/ml) combined with LTD_4_ (0.1 nM), the effect at 24 h was more potent than that of TGF-β1 or LTD_4_ alone (Figure 4).

**Figure 5:**
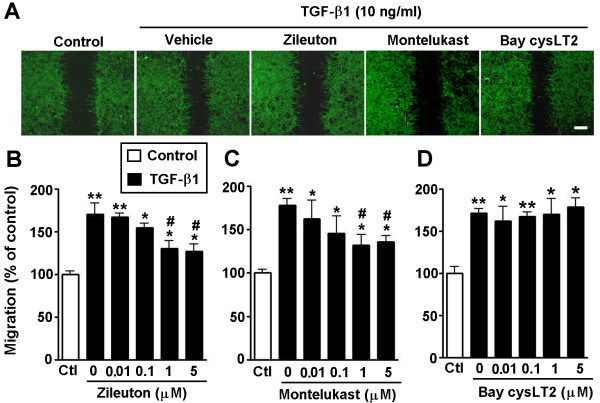
**Effects of a 5-LOX inhibitor and CysLT receptor antagonists on TGF-β1-induced migration in astrocytes.** (**A**) Photomicrographs showing TGF-β1 (10 ng/ml)-induced astrocyte migration 24 h after treatment with the 5-LOX inhibitor zileuton, the CysLT_1_R antagonist montelukast and the CysLT_2_R antagonist Bay cysLT2 (1 μM). Scale bar, 400 μm. (**B-D**) TGF-β1-induced migration inhibited by 0.01 to 5 μM zileuton (**B**) and montelukast (**C**), but not Bay cysLT2 (**D**). Data are reported as mean ± S.E.M.; *n* = 8; *P <0.05 and **P <0.01 compared with control; ^#^*P* <0.05 compared with TGF-β1 alone.

**Figure 6:**
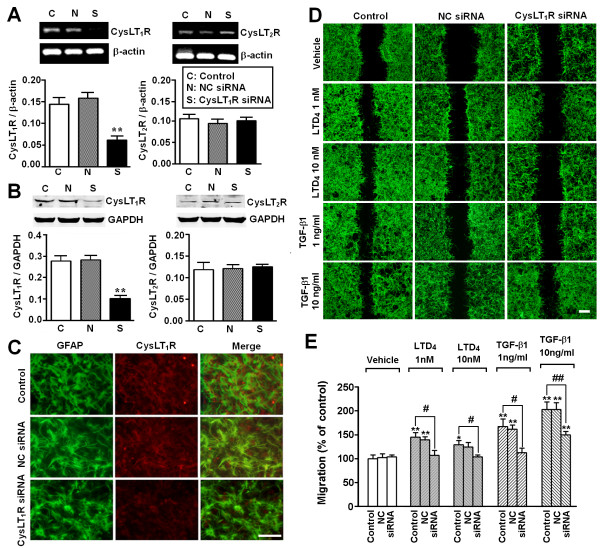
**Effect of CysLT**_**1**_**R siRNA on LTD**_**4**_**- and TGF-β1-induced migration in astrocytes.** (**A, B**) RT-PCR and Western blotting results showing inhibition of CysLT_1_R, but not CysLT_2_R, mRNA (**A**) and protein expression (**B**) by CysLT_1_R siRNA but not by negative control (NC) siRNA. (**C**) Double immunofluorescence staining showing inhibition of CysLT_1_R protein expression by CysLT_1_R siRNA in GFAP-positive astrocytes. (**D**) Photomicrographs showing that the astrocyte migration induced by LTD_4_ (1 and 10 nM) and TGF-β1 (1 and 10 ng/ml) was inhibited by CysLT_1_R siRNA (siRNA) but not by NC siRNA. (**E**) CysLT_1_R siRNA inhibited migration induced by LTD_4_ and TGF-β1. Data are reported as mean ± S.E.M.; *n* = 4 (A and B) or 8 (E); *P <0.05 and **P <0.01 compared with control; ^#^*P* <0.05 and ^##^*P* <0.01 compared with LTD_4_ or TGF-β1 alone. Scale bar, 200 μm (C) or 400 μm (D).

To confirm the roles of endogenous CysLTs and CysLT_1_R in TGF-β1-induced migration, we examined the effects of the 5-LOX inhibitor zileuton, the CysLT_1_R antagonist montelukast, and the CysLT_2_R antagonist Bay cysLT2 as well as CysLT_1_R siRNA. We found that the effect of 10 ng/ml TGF-β1 was attenuated by zileuton (1 and 5 μM, Figure 5A, B) and montelukast (1 and 5 μM, Figure 5A, C), but not by Bay cysLT2 (0.01 to 5 μM, Figure 5A, D). These results indicated that endogenously released CysLTs might activate CysLT_1_R, but not CysLT_2_R, to induce astrocyte migration and potentiate TGF-β1-induced migration. The involvement of CysLT_1_R was further confirmed by RNA silencing by transient transfection of CysLT_1_R siRNA into astrocytes. The siRNA (100 nM) significantly reduced the expression of CysLT_1_R mRNA (Figure 6A) and protein (Figure 6B, C), but the non-silencing negative control siRNA had no effect. CysLT_1_R siRNA significantly attenuated the effects of LTD_4_ (1 and 10 nM) and TGF-β1 (1 and 10 ng/ml) on astrocyte migration (Figure 6D, E). These results suggest that CysLT_1_R may be associated with LTD_4_- and TGF-β1-induced astrocyte migration.

### TGF-β1-Induced Activation of 5-LOX in astrocytes

**Figure 7:**
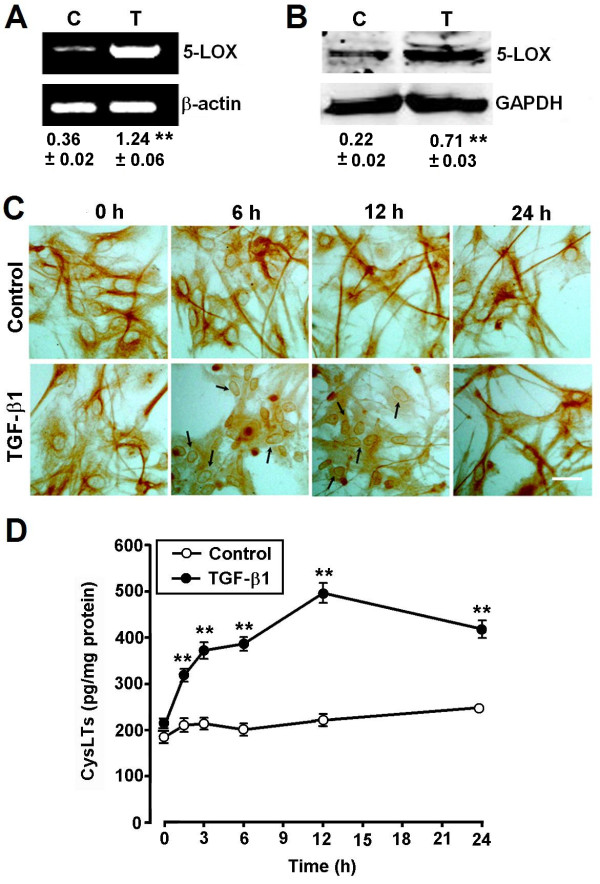
**TGF-β1 induces 5-LOX expression and translocation, and increases the production of CysLTs in astrocytes.** (**A, B**) RT-PCR and Western blotting results showing that 5-LOX mRNA (A) and protein expression (B) were increased in astrocytes after exposure to 10 ng/ml TGF-β1 for 24 h. Results are expressed as the ratios to β-actin (A) or GAPDH (B). C, control; T, TGF-β1. (**C**) Immunocytochemical examination reveals 5-LOX translocation to the nuclear envelope (arrows) in astrocytes after exposure to 10 ng/ml TGF-β1. Scale bar, 50 μm. (**D**) ELISA shows the production of CysLTs was significantly increased with a peak at 12 h after exposure to 10 ng/ml TGF-β1. Data are reported as mean ± S.E.M.; *n* = 4 (A and B) 8 (D); **P <0.01 compared with control.

To investigate the role of endogenous CysLTs, the 5-LOX metabolites, in TGF-β1-induced astrocyte migration, we determined 5-LOX expression in astrocytes. We found that TGF-β1 10 ng/ml significantly increased 5-LOX mRNA (Figure 7A) and protein expression (Figure 7B) 24 h after exposure. Immunocytochemical results showed that 5-LOX was translocated from the cytosol to the nuclear envelope 6 and 12 h after exposure to 10 ng/ml TGF-β1, and then recovered at 24 h (Figure 7C). We further determined the changes in enzymatic activity of 5-LOX by measuring its metabolites, CysLTs, in the culture medium. The levels of CysLTs increased from 1.5 h, peaked at 12 h, and were sustained over 24 h after exposure to 10 ng/ml TGF-β1 (Figure 7D). These findings revealed the involvement of 5-LOX and its metabolite CysLTs in the responses to TGF-β1.

### TGF-β1-regulated expression of CysLT receptor in astrocytes

**Figure 8:**
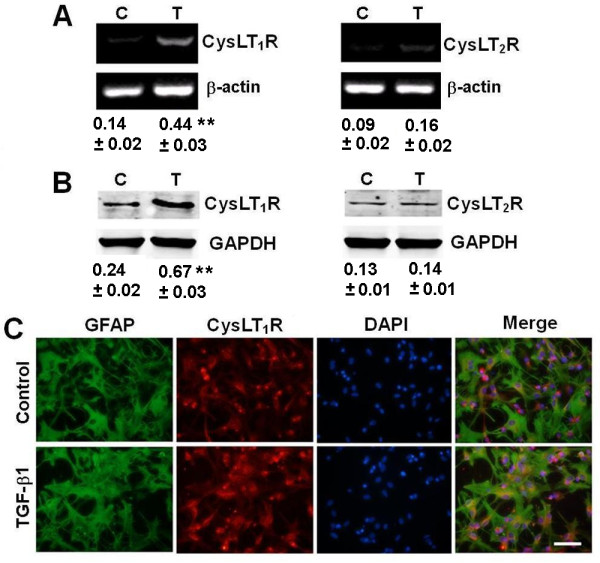
**Effect of TGF-β1 on expression of CysLT receptors in astrocytes.** (**A, B**) RT-PCR and Western blotting results showing that the mRNA (A) and protein expression (B) of CysLT_1_R, but not CysLT_2_R, in astrocytes increased after exposure to 10 ng/ml TGF-β1 for 24 h. Data are reported as mean ± S.E.M.; *n* = 4; **P <0.01 compared with control. Results are expressed as the ratios to β-actin (A) or GAPDH (B). C, control; T, TGF-β1. (**C**) Double immunofluorescence staining showing that TGF-β1 increased the expression of CysLT_1_R in GFAP-positive astrocytes. Scale bar, 100 μm.

**Figure 9:**
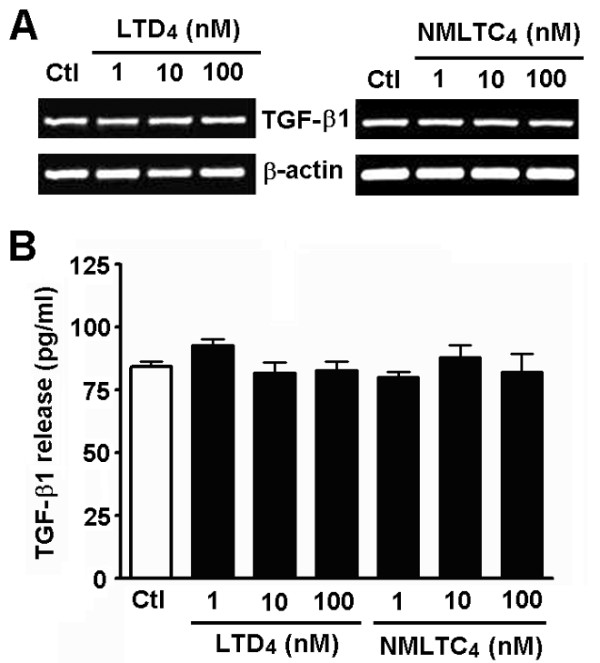
**Effects of LTD**_**4**_**and NMLTC**_**4**_**on TGF-β1 mRNA expression and TGF-β1 release in astrocytes.** (**A**), RT-PCR analysis showing mRNA expression of TGF-β1 after treatment with 1, 10 and 100 nM LTD_4_ or NMLTC_4_ for 24 h. There was no significant difference in TGF-β1 expression after treatment with LTD_4_ and NMLTC_4_ (*n* = 4 for each group, *P* >0.05). (**B**) TGF-β1 release in the culture supernatants after exposure to LTD_4_ and NMLTC_4_ measured by ELISA. Neither LTD_4_ nor NMLTC_4_ affected TGF-β1 expression and release. Data are reported as mean ± S.E.M.; *n* = 6.

Finally, we determined whether TGF-β1 regulates the expression of CysLT_1_R and CysLT_2_R mRNA and protein in astrocytes, and whether LTD_4_ regulates TGF-β1 expression and release. RT-PCR and Western blot showed weak expression of CysLT_1_R and CysLT_2_R in control astrocytes. Exposure to 10 ng/ml TGF-β1 for 24 h induced about three-fold increase in the mRNA (Figure 8A) and protein expression (Figure 8B) of CysLT_1_R, but did not significantly change the expression of CysLT_2_R. Immunofluorescence staining confirmed the enhancement of CysLT_1_R by TGF-β1 (Figure 8C). On the other hand, treatment with various concentrations of LTD_4_ or NMLTC_4_ for 24 h did not affect the TGF-β1 mRNA expression in astrocytes (Figure 9A) and its content in the culture medium (Figure 9B). Thus, TGF-β1 might up-regulate CysLT_1_R but is not regulated by LTD_4_.

## Discussion

In the present study, we revealed that TGF-β1-induced astrocyte migration is, at least in part, mediated by enhanced endogenous CysLTs through activation of CysLT_1_R. The evidence is that TGF-β1-induced astrocyte migration was potentiated by LTD_4_ but attenuated by a 5-LOX inhibitor and a CysLT_1_R antagonist, and TGF-β1 activated 5-LOX and increased CysLT_1_R expression. Our observations have confirmed the TGF-β1-induced migration of rat astrocytes as reported [6], and indicated another mechanism underlying TGF-β1-induced astrocyte migration in addition to the pathways through activation of the Smad family [47,48] or the ROS-dependent ERK/JNK-NF-κB pathway [6]. In addition, we found that both TGF-β1 and LTD_4_ did not alter astrocyte proliferation during 24 h. It has been reported that TGF-β1 inhibits astrocyte proliferation [47,49,50] and LTD_4_ induces the proliferation via activating CysLT_1_R [30]. This difference between these reported results and ours may result from different assessment timing [30] and methods [47,49,50]. However, in our experimental conditions, TGF-β1 and LTD_4_ regulate astrocyte migration rather than proliferation.

TGF-β1-induced astrocyte migration might be mediated by the CysLT signal pathway in at least two ways, that is, TGF-β1 potentiates the activity of both 5-LOX and CysLT_1_R. On one hand, TGF-β1 increased 5-LOX expression and induced its translocation to the nuclear envelope (Figure 7C), a key step for 5-LOX activation [51-53] and, thereby, increased the production of endogenous CysLTs (Figure 7D). Consistent with this, it has been reported that TGF-β1 induces 5-LOX expression in myeloid cell lines [54-58]. The notion is also supported by the finding that the TGF-β1 effect was inhibited by the 5-LOX inhibitor zileuton (Figure 5A). On the other hand, TGF-β1 potentiates the expression of CysLT_1_R, enhancing the activity of endogenously-produced or exogenous CysLTs as previously reported [37,39]. Therefore, one of the mechanisms underlying TGF-β1-induced astrocyte migration may be activation of endogenous 5-LOX/CysLT_1_R signals.

Here, we demonstrated that the receptor subtype that mediated the TGF-β1 effect was CysLT_1_R. The evidence was from the different effects of agonists and antagonists, and the effect of RNA interference. The non-selective agonist LTD_4_ induced a moderate migration of astrocytes at lower concentrations (0.1 to 10 nM), but not at the higher concentrations 100 nM (Figure 2A, C) and 1,000 nM (data not shown). This concentration-response relationship indicated that CysLT_1_R might mediate the effect of LTD_4_, because CysLT_1_R is activated at 1 to 10 nM while CysLT_2_R is activated at 100 to 1,000 nM in astrocytes [30]. This is also supported by the finding that the selective CysLT_2_R agonist NMLTC_4_[46] had no effect on astrocyte migration (Figure 3). With regard to receptor antagonism, the effect of TGF-β1 was attenuated by the CysLT_1_R antagonist montelukast but not by the CysLT_2_R antagonist Bay cysLT2. Bay cysLT2 is at least 100- to 500-fold more selective for CysLT_2_R versus CysLT_1_R; its pA_2_ value indicates that at least 5 μM would act on the CysLT_1_R [59,60]. Thus, lacking the effect of 5 μM Bay ctsLT2 in our study may be due to cell specificity and response difference. On the other hand, interference with CysLT_1_R siRNA inhibited both TGF-β1- and LTD_4_-induced astrocyte migration by down-regulating the expression of this receptor (Figure 6). These findings are consistent with reports that CysLT_1_R mediates the migration of other types of cells [31-36]. Therefore, CysLT_1_R is an important regulator of astrocyte migration in addition to its regulation of astrocyte proliferation [29,30].

The interaction between TGF-β1 and CysLTs was also investigated by determining the action of LTD_4_ or NMLTC_4_ on TGF-β1 expression and release. Unlike the action of TGF-β1 on the production of CysLTs and LTD_4_ effects, LTD_4_ or NMLTC_4_ affected neither TGF-β1 expression nor its release in astrocytes (Figure 9). This may depend on specific cell types because LTD_4_ induces TGF-β1 mRNA expression in human bronchial epithelial cells [61,62] and in fibroblasts from asthmatics [63], and LTC_4_ induces TGF-β1 production in airway epithelium [62] in a CysLT_1_R-dependent manner. Anyway, the effect of LTD_4_ on TGF-β1 in astrocytes remains to be further investigated, especially in animal models of chronic brain injury. Since both levels of TGF-β1 and CysLTs are increased after brain injury [24,64,65] and involved in glial scar formation [25,65,66]; which of them is determinant in glial scar formation should be clarified for their therapeutic implications. Herein, our results suggest that activation of the endogenous 5-LOX/CysLT_1_R signals might be an intermediate event in TGF-β1-regulated astrocyte migration, but not the initial event. Since TGF-β1 signaling is mainly modulated by Smad-dependent [67-75] and Smad-independent pathways [6,76-81], whether the regulation mode is mediated by the Smad or other pathways requires investigation.

Astrocyte migration is a critical step in the formation of a densely-packed glial scar [1,2], and TGF-β1 is closely associated with glial scar formation [64,66,82-84]. Thus, CysLT receptor antagonists or 5-LOX inhibitors may be beneficial in the prevention and attenuation of glial scar formation after brain injury. Actually, we have reported that the CysLT_1_R antagonist pranlukast attenuates glial scar formation in the chronic phase of focal cerebral ischemia in mice [28] and rats [25], and the 5-LOX inhibitor caffeic acid has this effect in rats with focal cerebral ischemia [85] and in mice with brain cryoinjury [86]. Moreover, montelukast inhibits the astrocyte proliferation induced by mild ischemia-like injury and low concentrations of LTD_4_[30]. The present study highlights the previous findings and clarifies the mode of action of endogenous CysLTs/CysLT_1_R in the critical step of glial scar formation.

**Figure 10:**
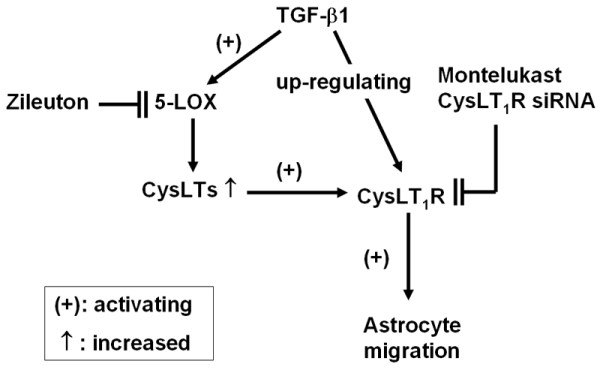
**Diagram showing the roles of TGF-β1 and 5-LOX/CysLT**_**1**_**R in induction of astrocyte migration.** TGF-β1 activates 5-LOX to produce CysLTs; the latter activates CysLT_1_R. Meanwhile, it also up-regulates CysLT_1_R expression, which enhances the activity of CysLT_1_R. The activated CysLT_1_R mediates TGF-β1-induced astrocyte migration.

In conclusion, in the present study we found that TGF-β1-induced astrocyte migration is, at least in part, mediated by enhanced endogenous CysLTs through activating up-regulated CysLT_1_R (Figure 10). These findings indicate that the interaction between the cytokine TGF-β1 and pro-inflammatory mediators (CysLTs) are involved in the regulation of astrocyte function relevant to glial scar formation. However, the detailed mechanisms underlying this interaction need investigation.

## Abbreviations

5-LOX, 5-lipoxygenase; CNS, central nervous system; CysLT1R, cysteinyl leukotriene receptor 1; CysLT2R, cysteinyl leukotriene receptor 2; CysLTs, Cysteinyl leukotrienes; FBS, fetal bovine serum; GAPDH, glyceraldehyde 3-phosphate dehydrogenase; GFAP, glial fibrillary acidic protein; LTD4, leukotriene D4; MFI, mean fluorescence intensity; NMLTC4, N-methyl leukotriene C4; PBS, phosphate-buffered saline; siRNA, small interfering RNA; TGF-β1, transforming growth factor- β1.

## Competing interests

The authors have no competing interests.

## Authors’ contributions

XQH designed the study, performed the main parts of the experiments, analyzed the data, and prepared the manuscript. XYZ performed the immunofluorescence staining experiments and prepared the figures. XRW performed the cell migration experiments and analyzed the data. SYY performed the RT-PCR experiments and analyzed the data. SHF, YBL and WPZ contributed to the design of the study, to interpretation of the results and to the writing of the manuscript. EQW made essential contributions to the design of the study and interpretation of the results, and completed the manuscript. All authors read and approved the final manuscript.
